# Prostitution in London, &c.

**Published:** 1839-04

**Authors:** 


Art. XIV.—*Prostitution in London; with a Comparative View of that of Paris and New York, as illustrative of the Capitals and Large Towns of all Countries; and proving Moral Depravation to be the most fertile source of Crime, and of Personal and Social Misery. Illustrated by numerous Plates, showing the Diseases.* By Michael Ryan, m.d.—*London*, 1839. 12mo. pp. 447.

After carefully examining this book, and reading as many of its pages as disgust would permit, we do not hesitate to pronounce it disgraceful to our literature. It is a wretched compilation, almost as low in its literary qualities as it is loathsome in its spirit and in its details. We thought it impossible that anything could go beyond the work *On Marriage*, reviewed by us in a former Number; but the volume before us has shown that even "in the lowest deep a lower deep" of obscenity and filth remained to be explored. The notice which we took of Duchatelet's work *On Prostitution in Paris*, and the commendation we bestowed on it, suffice to show that we have no wish to eschew the consideration of such subjects, and that we do not regard them as unimportant, or unbecoming the dignity or decency of medicine. Had the present work given us similar information respecting London, we should have received it with respect and even thankfulness. But the volume before us is in character and tendency entirely different. With the exception of a bad abridgment of Duchatelet's book in the commencement (the only portion of the volume, in our opinion, of the slightest value), we have no record of statistical facts of the least importance, and scarcely a particle of information respecting London that was not known to every reader of the morning newspapers, or of the reports of the excellent societies established in the metropolis for the suppression of vice and immorality. This circumstance we wish particularly to impress on the minds of our readers, lest they might be seduced, by the prospect of important information, to overleap or wade through the polluting barriers by which they might expect, from former experience, to find it surrounded. The greater portion of the volume is filled with details of the most disgusting kind, selected from the commonest sources of impurity, crowded one upon another, pell-mell, page after page, without any necessary connexion with the professed subject of the work, unredeemed by the slightest tincture of utility, and only calculated—we do not say intended—to pander to the vilest and most depraved tastes.

If it were worth while to criticise a production which has no claim either to scientific research or literary merit, we might begin with its title. To show that *moral* depravation is the most fertile source of crime may not be very difficult: many, however, will have doubts as to whether it be the most fertile source of personal and social misery. At any rate a physician worthy of the title might, one would think, have learned that personal and social misery are also most fertile causes of moral depravation. It would do no dishonour to the authors of indecent books if they had such a plea to offer. Yet, even in that case, we should be inclined to take Dr. Samuel Johnson's view of the matter; and if assured by such apologists that "they *must* live," to reply, that we did not see the *necessity* for *that*. Our readers may well suppose that the task of noticing such works as that before us is none of the pleasantest. But neither this consideration, nor the effrontery with which such outrages on decency are defended, on the score of their being profitable, must avert us from the performance of a duty devolving upon us as journalists, or make us forget what is due to the honorable profession under the sanction of the honorable titles of which such abominations are shamelessly committed.

